# Conscious sedation in non comatose patients submitted to intra - arterial thrombolysis: our experience

**DOI:** 10.1186/2197-425X-3-S1-A990

**Published:** 2015-10-01

**Authors:** Y Valzani, A Marudi, S Baroni, A Zini, S Vallone, E Bertellini

**Affiliations:** Universita degli Studi di Modena e Reggio Emilia, Modena, Italy; Nuovo Ospedale Civile Sant'Agostino Estense, Modena, Italy

## Introduction

Recent studies have demonstrated that general anesthesia worsen outcome of patients affected by acute ischemic stroke (AIS) who underwent intra arterial thrombolysis [1]. General anesthesia (GA) guarantees adequate oxygenation and prevents pneumonia aspiration. On the other hand general anesthesia induces hypotension worsening brain injury. Futhermore it does not allow to verify early recovery of neurological deficits during the procedure.

Conscious sedation (CS) can represent a valid and reliable option in non comatose patients suffering from AIS and candidated to endovascular reperfusion procedure [2].

## Objectives

To show our experience in CS during intra arterial reperfusion.

## Methods

We collected data from patients affected by AIS who underwent intra arterial thrombolysis (pharmacological thrombolysis and mechanical thrombectomy) admitted in our hospital from January 2014 to December 2014 and the anesthesia type. We recorded demographics data, Glasgow Coma Scale score at admission and lenght hospital stay. Data were expressed as mean ± standard deviation.

## Results

59 affected by AIS were admitted to Our hospital and submitted to endovasculare recanalization procedure. Male to female ratio was 1:1, mean age 59 ± 4, mean Glasgow Coma Scale score (GCS) at the admission was 11 ± 2, mean duration of procedure was 58 ± 16 minutes.

General anesthesia was performed in 12 patients and conscious sedation in 47 patients.

Patients were submitted to GA because of patients' anxiety or rapid worsening of neurological condition.

6 patients submitted to GA died in intensive care unit becasue of neurological complications.

Mean hospital lenght of stay was 8 ± 4 days for patients underwent CS.

Mean hospital lenght of stay was 18 ± 7 days for patients underwent GA.

## Conclusions

CS can reduce lenght of stay in hospital and mortality of non comatose patients who undergo intra arterial reperfusion in comparison to general anesthesia [3]. Futher investigations are necessary to confirm our results.
